# Prospective randomized controlled trial of the safety and feasibility of a novel mesenchymal precursor cell therapy in hypoplastic left heart syndrome

**DOI:** 10.1016/j.xjon.2023.09.031

**Published:** 2023-10-01

**Authors:** Rachel E. Wittenberg, Kimberlee Gauvreau, Jonah Leighton, Melinda Moleon-Shea, Kenneth M. Borow, Gerald R. Marx, Sitaram M. Emani

**Affiliations:** a Harvard Medical School, Boston, Mass; b Department of Cardiology, Boston Children's Hospital, Boston, Mass; c Department of Cardiac Surgery, Boston Children's Hospital, Boston, Mass; d Mesoblast, Inc, New York, NY

**Keywords:** hypoplastic left heart syndrome, biventricular conversion, left ventricular recruitment, stem cell therapy, congenital heart disease, regenerative medicine, clinical trial

## Abstract

**Objective:**

To assess the safety and feasibility of low-dose, novel, allogenic mesenchymal precursor cell (MPC) therapy as an adjunct to left ventricular (LV) recruitment for patients with hypoplastic left heart syndrome (HLHS) and borderline left ventricles. MPC injections into the hypoplastic left ventricle may stimulate neovascularization and beneficial LV remodeling and may improve the likelihood of achieving biventricular (BiV) or 1.5 ventricle (1.5V) circulation.

**Methods:**

Children <5 years with prior single ventricle palliation undergoing LV recruitment surgery at a single center were randomized to MPC injections into the LV endocardium/papillary muscles (MPCs) or standard-of-care (controls) and followed for 24 months. The primary endpoint was safety, including (serious) adverse events (S/AEs), and panel reactive antibodies (PRAs). Secondary endpoints included BiV/1.5V conversion and LV size and function.

**Results:**

Nineteen subjects were enrolled, including 9 MPC recipients and 10 controls. Fourteen patients (74%) had >1 AE, and 2 patients had SAEs, both deemed unrelated to the trial product. AE severity and frequency were similar in the 2 groups. Baseline PRA levels were high, with no difference between the groups at 12 months. The overall probability of BiV/1.5V conversion was 0.16 (95% confidence interval [CI], 0.05 to 0.41) at 12 months and 0.52 (95% CI, 0.31 to 0.77) at 24 months. For patients with imaging data at both time points, increases in LV volumes from baseline to 12 months were larger in the MPC group by 3-dimensional echocardiography and cardiac magnetic resonance imaging. For children who successfully underwent BiV conversion (n = 12), full BiV conversion was achieved at 24 months in 5 of 5 (100%) MPC-treated children compared with 4 of 7 (57%) controls.

**Conclusions:**

MPC injections were considered safe and feasible in HLHS patients. More than 50% of subjects underwent BiV/1.5V conversion within 2 years. Larger trials are needed to investigate the therapeutic potential of MPCs in this population.

## Graphical Abstract

**Figure:**
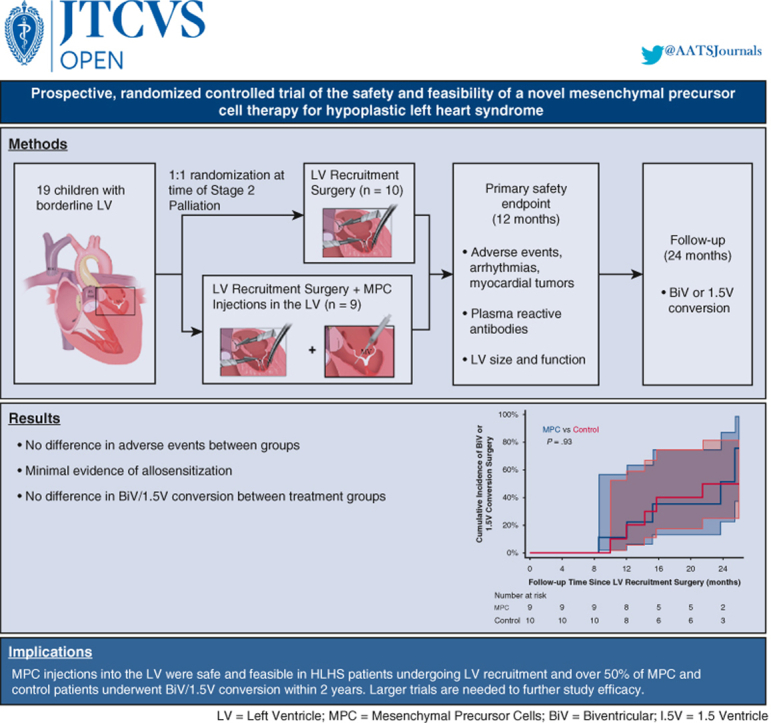

**Figure:**
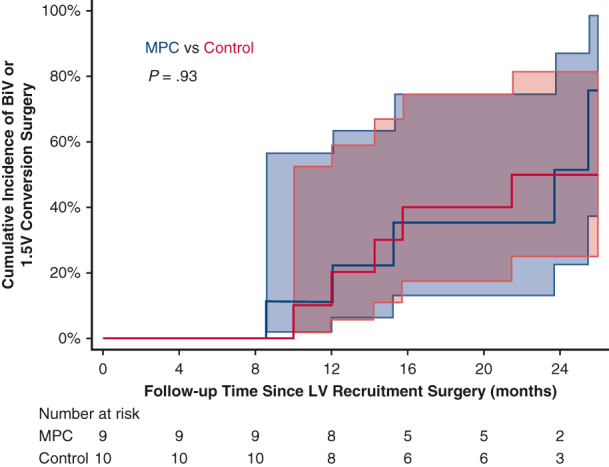
BiV/1.5V conversion in patients in a randomized controlled trial of LV MPC injections.

**Central Message**

A novel mesenchymal precursor cell therapy was safe and feasible in pediatric patients with HLHS undergoing staged LV recruitment surgery. More than 50% achieved biventricular circulation within 2 years.

**Perspective**

Choosing single ventricle palliation or biventricular conversion is challenging in patients with HLHS and a borderline left ventricle. Stem cell therapy combined with left ventricular (LV) recruitment procedures to progressively increase LV volume loading may stimulate LV neovascularization and beneficial remodeling, increasing the likelihood of achieving successful BiV/1.5V circulation. We demonstrate the safety and feasibility of this approach.

See Discussion on page 673.

Hypoplastic left heart syndrome (HLHS) is a spectrum of severe congenital heart disease (CHD) characterized by underdeveloped left-sided cardiac structures. Single ventricle palliation through the Fontan operation is standard of care but is associated with long-term morbidity secondary to right heart failure, including renal failure, hepatic steatosis, arrhythmias, and protein-losing enteropathy.^1^ Attempts at early biventricular (BiV) conversion may carry a higher perioperative risk and lead to long-term diastolic dysfunction in some patients.^2^^,^^3^ An intermediate option is staged left ventricular (LV) recruitment with initial stage 1 palliation, followed by a superior cavopulmonary anastomosis (Glenn) surgery and surgical maneuvers to gradually promote volume loading of the hypoplastic left ventricle, including aortic and mitral valvuloplasty, resection of endocardial fibroelastosis (EFE), and atrial septal defect restriction.

Following staged recruitment, patients with sufficient LV growth are candidates for BiV or reverse 1.5 ventricle (1.5V) repair.^4^ Patients with inadequate LV growth usually progress to a Fontan palliation. These strategies are helpful in some patients; however, a majority of patients with a borderline left ventricle are still unable to achieve successful biventricular conversion.^3^^,^^5^

Mesenchymal lineage cells (MLCs), are nonhematopoietic, multipotent stem cells with anti-inflammatory, angiogenic, and immunologic properties.^6^^,^^7^ Intracardiac MLC injections have demonstrated safety and potential efficacy for adults with myocardial infarction (MI),^8^, ^9^, ^10^, ^11^, ^12^ ischemic or nonischemic cardiomyopathy,^13^ and heart failure.^14^ Although there are some studies of MLCs in CHD,^15^, ^16^, ^17^, ^18^ there is a lack of randomized control trials for patients with a borderline left ventricle. In pediatric patients undergoing LV recruitment, MLC injections into the hypoplastic left ventricle may stimulate local tissue neovascularization and beneficial LV remodeling, improving the likelihood of achieving increases in LV volumes and subsequent BiV or 1.5V circulation.

We conducted this prospective single-center, modified double-blind, randomized controlled trial of allogenic mesenchymal precursor cell (MPC) injections versus standard-of-care surgical treatment without MPC injections (controls) in children with HLHS undergoing LV recruitment. The goal of the study was to assess the safety and feasibility of intraoperative MPC injections, with the secondary endpoint of LV size and function at 1 year following MPC administration. The study is summarized in a graphical abstract in Figure 1.

**Figure 1:**
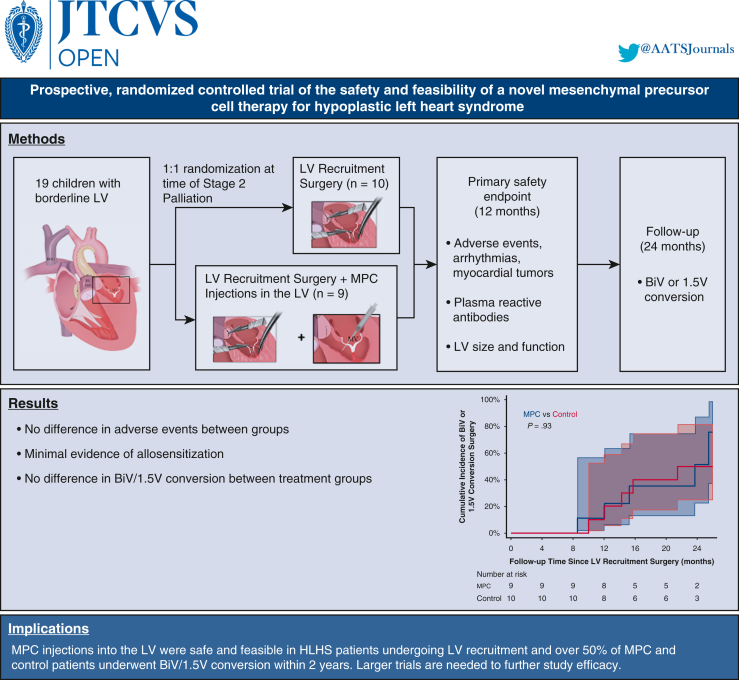
Graphical abstract. *LV*, Left ventricle; *MPC*, mesenchymal precursor cell; *BiV*, biventricular; *1.5V*, 1.5 ventricle.

## Methods

### Study Enrollment

This clinical trial conformed to the Declaration of Helsinki and was approved by the Boston Children's Hospital Institutional Review Board (IRB-P00020575; August 8, 2016). The trial was registered at ClinicalTrials.gov (identifier NCT03079401). Written informed consent was obtained publication of study data for all subjects. Patients age ≤5 years with a diagnosis of HLHS, HLHS with variants, unbalanced atrioventricular (AV) canal, or borderline LV with an LV *z*-score of −10 to +2 at a single center were eligible for enrollment if they were undergoing bidirectional Glenn surgery (BDG) and LV recruitment or had a history of BDG and were undergoing LV recruitment alone. All patients were required to have previously undergone stage 1 palliation, pulmonary artery banding, or a hybrid palliation procedure. Patients with prior Fontan surgery were excluded. Patients were also excluded if they had active or prior malignancy, a myocardial tumor, a history of aortic or mitral atresia, or previous high-grade ventricular arrhythmias; had an allergy to dimethyl sulfoxide, mouse, or cow products; had received any prior stem cell therapy for cardiac repair; or had potentially received any investigational cell-based therapy within 6 months of randomization.

### Randomization

Enrolled subjects were randomized 96 hours prior to surgery to receive either LV recruitment surgery and MPC injections (MPC group) or LV recruitment surgery without MPC injections (control group) in a 1:1 ratio, using block randomization with a block size of 4. The trial was planned for enrollment of 24 subjects, based on similarly designed phase I trials in the field, feasibility of enrollment, and MPC availability. However, partway through the trial, the manufacture and release of MPCs was curtailed, limiting the total number of randomized subjects to 19.

The study used a modified double-blind design. Patients/families, research staff including the trial biostatistician, imaging technologists, and clinical staff outside of the operating room were all blinded to randomization assignment until the last subject completed 12 months of follow-up. No placebo injections were administered, and all MPC injections were completed by the principal investigator, irrespective of primary surgeon. Thus, the study's research nurse, operating room staff, and principal investigator could not be blinded because they were involved in shipping, storing, and administrating the MPCs.

### MPC Administration

Proprietary STRO-3 selected allogenic mesenchymal lineage stem cells derived from the bone marrow of 3 young, healthy adult donors and expanded ex vivo were procured from Mesoblast. These “immune privileged” MPCs lack specific cell surface costimulation molecules and so should not generate a significant antibody response in unmatched patients.

**Figure 2:**
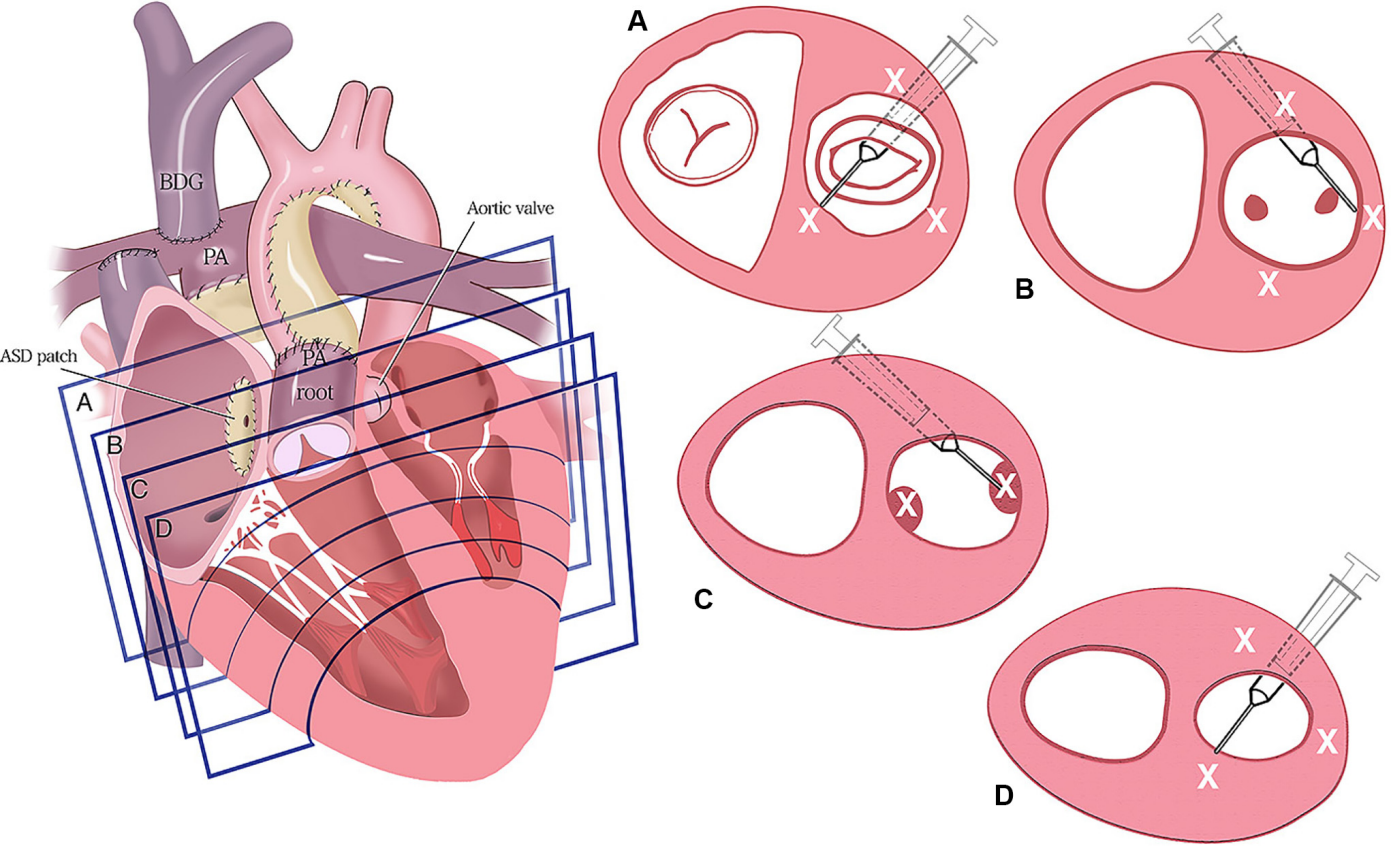
Intraoperative mesenchymal precursor cell (*MPC*) injection sites in a hypoplastic left ventricle. For patients in the MPC group, a dose of approximately 20 × 10^6^ MPCs was delivered using a 23- to 25-gauge needle into each of the 2 left ventricular (*LV*) papillary muscles and throughout the upper (A), mid (B and C), and apical (D) regions of the LV endocardium following completion of all planned surgical procedures (maximum volume, 550 μL). *BDG*, Bidirectional Glenn; *PA*, pulmonary artery; *ASD*, atrial septal defect.

A low dose of approximately 20 million MPCs was chosen based on previous adult studies,^14^^,^^19^, ^20^, ^21^ proportional to LV mass in HLHS patients. MPCs were delivered to the LV endocardium under direct surgical visualization with a 23- to 25-gauge needle, using approximately 11 injections of 50 μL each. One injection was performed into each of the two LV papillary muscles, with the remaining nine injections distributed throughout the upper, mid, and apical regions of the LV endocardium to ensure diffuse distribution throughout the LV (Figure 2).

### Follow-up

Patients were followed for 1 week postsurgery or until hospital discharge. Subsequent preplanned trial visits and clinical testing were designed to occur at 1 to 4 weeks, 3 to 6 months, 12 months, and 24 months following surgery. Additional monthly phone calls were conducted between 6 and 12 months and at 18 months following surgery. The timing and duration of follow-up were based on the anticipated time frame for safety events and BiV/1.5V conversion. Adverse events (AEs) and serious adverse events (SAEs) were recorded throughout the study period. A Data Safety and Monitoring Board reviewed AEs and other trial data at preplanned intervals, with all SAEs undergoing immediate review.

Telemetry review was conducted every 8 hours for 24 hours following surgery. If there were no arrhythmias, telemetry was continued daily until day 7 or hospital discharge. Medical history, occurrence of AEs, and medications were reviewed at all in-person and telephone study visits. Physical examinations were performed at all in-person study visits. Echocardiograms and electrocardiograms were analyzed at baseline, 1 to 4 weeks, 3 to 6 months, 12 months, and 24 months. Cardiac MRI and cardiac catheterization were performed at baseline and 12 months. Two blinded readers completed all LV measurements by 3-dimensional (3D) echocardiography while multiple blinded readers completed the 2-dimensional (2D) echocardiography and cardiac MRI interpretations. Plasma reactive antibodies (PRA) were drawn at baseline, prior to hospital discharge, and at 12 and 24 months postdischarge, and donor-specific antibodies (DSA) were analyzed for all patients who received MPCs and had PRA ≥5%.

### Statistical Analysis

Baseline patient and clinical characteristics were summarized for the overall cohort and by group. Frequency and percentage are reported for categorical variables, and median with interquartile range are reported for continuous variables. The proportion of patients experiencing an AE were compared using the Fisher exact test. For imaging data, changes from baseline to 12-month follow-up were compared between groups using the Wilcoxon rank-sum test. Comparisons of changes in echocardiography and PRA data across multiple time points were performed using mixed models. The cumulative incidence of BiV/1.5V conversion surgery after LV recruitment surgery was estimated using the Kaplan-Meier method and compared between groups using the log-rank test. The Greenwood method was used to estimate 95% confidence intervals (CIs). All hypothesis tests were conducted at the 0.05 significance level. Analyses were performed in Stata version 16.0 (StataCorp).

## Results

Overall results are on a primary intention-to-treat (ITT) basis. In some cases, treated patient analyses differed from ITT analyses.

### Demographic Data

**Figure E1:**
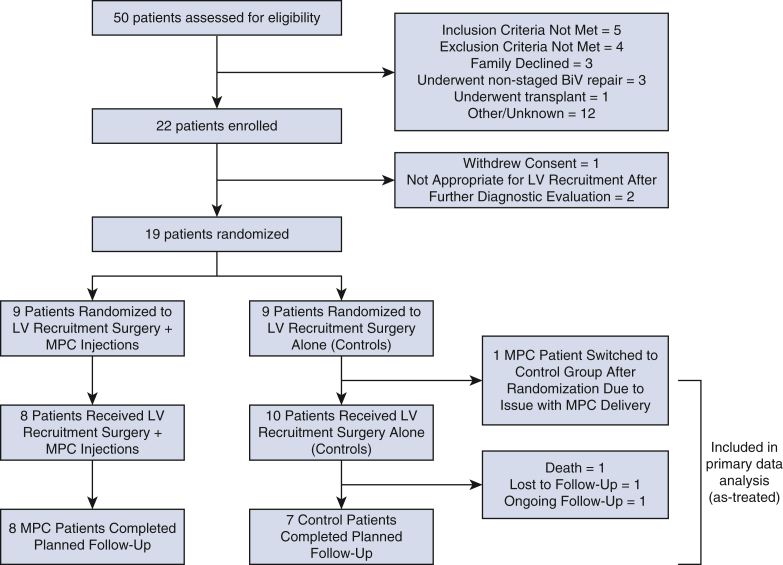
CONSORT flow diagram showing trial enrollment, randomization, and follow-up. *LV*, Left ventricular; *MPC*, mesenchymal precursor cell.

Nineteen patients were randomized between March 2018 and October 2021. Nine patients were randomized to MPC injections, and 10 patients were randomized as controls (standard-of-care surgical treatment with no injections). One patient was randomized to MPC treatment but did not receive cells owing to shipment issues. This patient is included as initially randomized in the MPC group in the primary intention-to-treat analyses. One patient in the control group was lost to follow-up prior to their 12-month visit. Enrollment, randomization, and follow-up are summarized in Figure E1.

Demographic and selected baseline clinical information for the trial participants were similar between the MPC and control groups (Table 1). Fifteen of the 19 patients (79%) had HLHS, and 4 patients (21%) had an unbalanced AV canal. Three patients had undergone LV recruitment procedures (eg, EFE resection) during prior surgeries. Ten patients (53%) had undergone previous BDG, whereas the remaining patients had a BDG during LV recruitment surgery at the time of trial enrollment. Additional procedures performed during LV recruitment surgery at the time of trial enrollment included retention or creation of a systemic–pulmonary shunt (79%), aortic or mitral valve repair (68%), and atrial septal defect restriction (95%), with no significant differences between groups.

**Table 1:** Demographic information and select baseline clinical characteristics, by treatment assigned

Characteristic	Total (N = 19)	MPC group (N = 9)	Control group (N = 10)
Age at randomization mo, median (IQR)	11.5 (5.8-28.4)	17.9 (6.1-27.0)	9.0 (4.6-28.4)
Female sex, n (%)	5 (26)	3 (33)	2 (20)
Race/ethnicity, n (%)			
White	18 (95)	8 (89)	10 (100)
Hispanic	1 (5)	1 (11)	0 (0)
Cardiac diagnosis, n (%)			
HLHS/HLHS variant	15 (79)	7 (78)	8 (80)
Unbalanced AVC	4 (21)	2 (22)	2 (20)
Any previous cardiac surgery, n (%)	19 (100)	9 (100)	10 (100)
LV recruitment surgery prior to trial enrollment	3 (16)	1 (11)	2 (20)
BDG prior to trial enrollment	10 (53)	5 (56)	5 (50)
Weight, kg, median (IQR)	8.7 (6.4-12.6)	8.7 (6.5-12.6)	8.6 (6.4-11.7)
Height, cm, median (IQR)	74 (62-88)	74 (65-88)	71 (62-88)
LVEDV indexed to BSA (mL/m^2^) by 3D echocardiography, median (IQR)	19.9 (15.1-28.3)	17.4 (14.1-21.9)	27.1 (16.4-33.8)
LV mass indexed to BSA (g/m^2^) by 3D echocardiography, median (IQR)	19.1 (17.2-25.9)	18.5 (16.5-24.0)	20.6 (18.2-29.0)

*MPC*, Mesenchymal precursor cell; *IQR*, interquartile range; *HLHS*, hypoplastic left heart syndrome; *AVC*, atrioventricular septal defect; *LV*, left ventricular; *BDG*, bidirectional Glenn; *LVEDV*, left ventricular end-diastolic volume; *BSA*, body surface area; *3D*, 3-dimensional.

The median age at randomization was 11.5 months (IQR, 5.8-28.4 months), 5 patients (26%) were female, and 18 patients (95%) identified as white. At baseline, the median LV end-diastolic volume (LVEDV) indexed to body surface area (BSA) using 3D echocardiography was 19.9 mL/m^2^ (IQR, 15.1-28.3 mL/m^2^), and the median LV mass indexed to BSA was 19.1 g/m^2^ (IQR, 17.2-25.9 g/m^2^).

### Adverse Events

There were 2 SAEs over the course of the trial. One control patient sustained a bradycardic arrest on postoperative day 8 in the setting of a transient rise in Glenn pressures and oxygen desaturation. The patient had prompt return of spontaneous circulation and no long-term sequela. One MPC patient died 21 months after surgery following a complex clinical course with biventricular systolic failure necessitating placement of a, LV assist device, extracorporeal membrane oxygenation, and prolonged hospitalization. Both SAEs were evaluated by the study's Data Safety and Monitoring Board and deemed unlikely to be related to the trial product or administration. No cardiac tumors, intracardiac hematomas, or perioperative ventricular arrhythmias were detected in any trial participants.

Overall, 74% of patients had at least 1 AE during follow-up, with no significant difference between the MPC and control groups (*P* = .30). The median number of AEs per patient per year of follow-up was not statistically different between the groups, with 1.6 events/patient/year (IQR, 0.8-3.8) in the MPC group and 1.1 events/patient/year (IQR, 0-3.6) in the control group (*P* = .39). There was no significant difference in the proportion of patients with AEs or the highest AE severity rating between the groups (*P* = .17). Finally, there was no significant difference between the groups in the time from LV recruitment surgery to first recorded AE (*P* = .18) (Table 2).

**Table 2:** Frequency and severity of AEs throughout 24 months of follow-up, by treatment assigned

Parameter	Total (N = 19)	MPC group (N = 9)	Control group (N = 10)	*P* value
Any AE during follow-up, n (%)	14 (74)	8 (89)	6 (60)	.30
Total AEs, n (%)	68	39	29	—
Events per patient, median (IQR)	3 (0-4)	3 (1-5)	2 (0-4)	.32
AEs per year of follow-up, median (IQR)	1.6 (0-3.8)	1.6 (0.8-3.8)	1.1 (0-3.6)	.39
Highest event severity, n (%)				.17
None	5 (26)	1 (11)	4 (40)	
Mild	5 (26)	4 (44)	1 (10)	
Moderate	4 (21)	3 (33)	1 (10)	
Severe	5 (26)	1 (11)	4 (40)	
Cumulative incidence of AEs, %				.18
2 mo	57.9	77.8	40.0	
6 mo	68.4	88.9	50.0	
12 mo	73.7	88.9	60.0	

*MPC*, Mesenchymal precursor cell; *AE*, adverse event; *IQR*, interquartile range.

### HLA Sensitization

**Figure 3:**
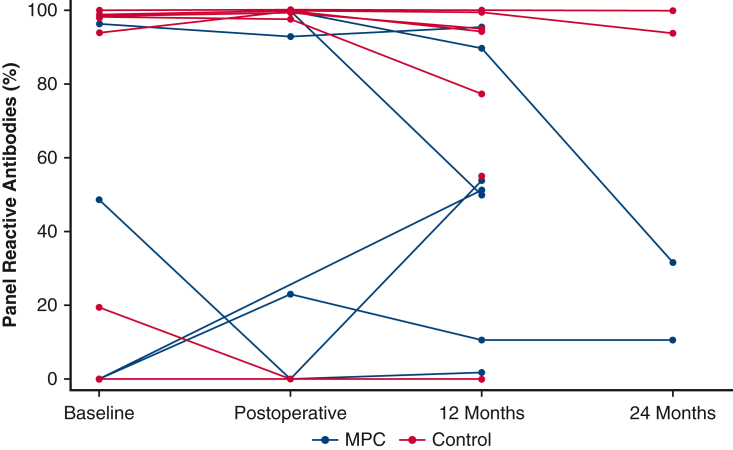
Plasma reactive antibody (*PRA*) levels at baseline and throughout follow-up, by treatment assigned. Percent PRA levels (representing HLA sensitization) are shown at baseline and throughout up to 2 years of preplanned follow-up, by treatment assigned. Two patients had an increase in PRAs of >10% at any point during follow-up. Patients with missing PRA levels at any time point are shown, with missingness accounted for in the mixed models. *MPC*, Mesenchymal precursor cell.

Both the MPC and control groups had a bimodal PRA distribution, with overall high HLA sensitization. At baseline, 11 of 18 patients (61%) had PRA >10%, and 9 (50%) had PRA >90%. There was no difference in PRA% between groups across all time points (*P* = .73). The majority of subjects maintained relatively constant PRA levels throughout follow-up (Figure 3). Only 2 patients had a PRA increase of >10% at any point over the course of follow-up. One MPC patient had an increase in PRA from 0% at baseline to 51.4% at 12 months, with no subsequent measurements. One patient had an increase in PRA from 0% at baseline to 23.1% postoperatively, which then stabilized to 10.6% at 12 months and 24 months. Of note, this patient is included as an MPC patient in the intention-to-treat analysis in Figure 3 but represents the subject who was crossed over to the control group after randomization and did not receive MPCs.

Although DSA were present in some highly sensitized MPC patients at baseline, in only 2 instances did MPC patients “acquire” new DSA over the course of study follow-up. One additional DSA was present in the postoperative period and at 12 months in a patient who was highly sensitized (PRA 99.99%) with 5 additional DSA present at baseline. One new DSA was present at 12 months in a patient who was unsensitized at baseline.

### Full Biventricular and 1.5 Ventricle Conversion

**Figure 4:**
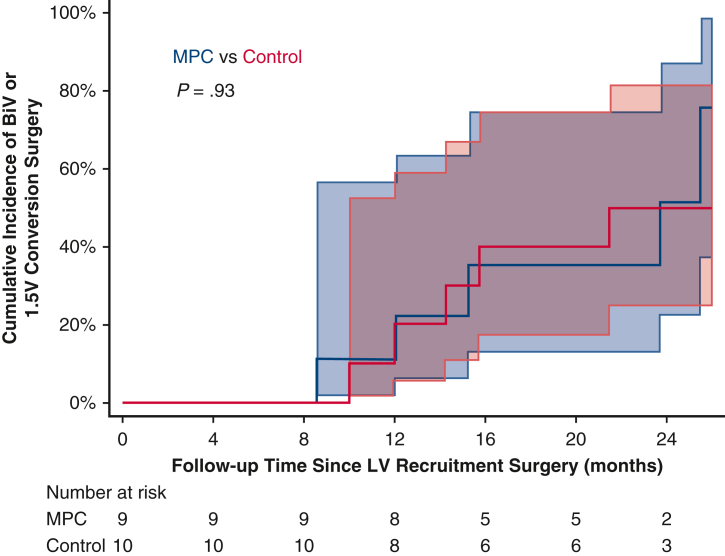
Time from left ventricular (*LV*) recruitment surgery to biventricular (*BiV*) or reverse 1.5 valve (*1.5V*) conversion surgery within 2 years of preplanned follow-up, by treatment assigned. Shaded areas represent 95% confidence intervals.

Overall, 9 patients (47%) underwent complete BiV conversion, and 3 patients (16%) underwent 1.5V conversion prior to the final 2-year follow-up. Six patients (32%) remained on a single ventricle palliation strategy at 2-year follow-up, and 1 patient (5%) had a single ventricle palliation strategy but has not yet completed follow-up. No patients proceeded to Fontan surgery prior to 2-year follow-up. Of the 6 patients in the MPC group who underwent BiV or 1.5V conversion, 5/6 (83%) underwent complete BiV conversion. Of the 6 patients in the control group who underwent BiV or 1.5V conversion, 4/6 (67%) underwent complete BiV conversion. There were no significant differences between the MPC and control groups in time to BiV/1.5V conversion (*P* = .93). In the overall cohort, the probability of BiV/1.5V conversion was 0.16 (95% CI, 0.05-0.41) at 12 months and 0.52 (95% CI, 0.31-0.77) at 24 months (Figure 4). For MPC-treated children, 100% (5 of 5) were able to undergo the preferred full BiV conversion. No MPC-treated patients underwent the 1.5V surgical procedure. In contrast, for the control children, 57% (4 of 7) had the full BiV conversion whereas 43% (4 of 7) underwent the more limited 1.5V surgery.

### Imaging and Catheterization Data

Baseline data were similar in the MPC and control groups for all imaging modalities. There were significant increases in LVEDV, LVESV, and LV mass from baseline to 12 months in both the MPC and control groups. By 3D echocardiography, the MPC group had significantly larger increases in LVEDV (*P* = .020) and LVESV (*P* = .009) indexed to BSA from baseline to 12 months compared to the control group. At 12 months, the median LVEDV in the MPC group was 81.7 mL/m^2^ (IQR, 56.7-107.8 mL/m^2^), compared to 41.8 mL/m^2^ (IQR, 37.3-60.8 mL/m^2^) in the control group. The median LVESV in the MPC group was 28.1 mL/m^2^ (IQR, 20.8-40.8 mL/m^2^) compared to 12.9 mL/m^2^ (IQR, 11.3-23.9 mL/m^2^) in the control group. LV mass indexed to BSA and LV ejection fraction increased significantly from baseline to 12 months in the overall cohort, but there was no significant difference between the groups (*P* = .85 and .34, respectively).

**Figure 5:**
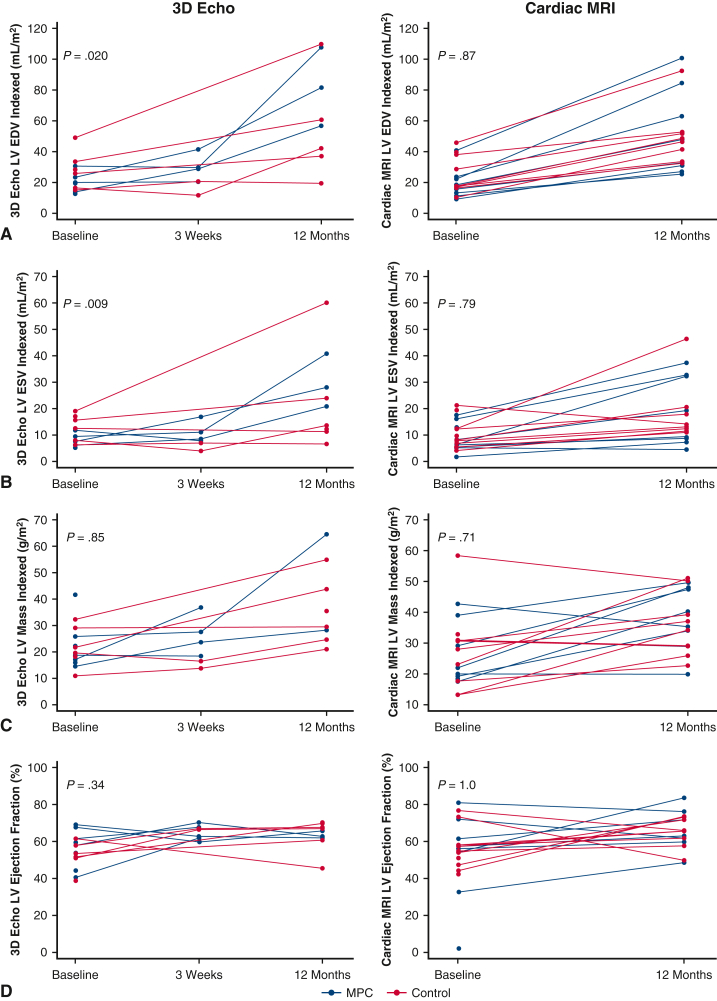
Change from baseline to 12 months in left ventricular (*LV*) size and function by 3-dimensional (*3D*) echocardiography and cardiac magnetic resonance imaging (*MRI*), by treatment assigned. Change in LV end-diastolic volume (*LVEDV*) (A), LV end-systolic volume (*LVESV*) (B), LV mass (C), and LV ejection fraction (D) from baseline to primary efficacy analysis at 1 year, by treatment assigned: 3D echocardiography (*left*) and cardiac MRI (*right*). The numbers of patients with 3D echocardiography and cardiac MRI data at each time point are shown in Table 3. Missing data are not shown and did not contribute to median calculations and comparison between groups. *MPC*, Mesenchymal precursor cell.

Cardiac MRI revealed no significant difference between the groups in changes in LVEDV (*P* = .87) or LVESV (*P* = .79) from baseline to 12 months. The median LVEDV at 12 months was 40.4 mL/m^2^ (IQR, 29.2-73.9 mL/m^2^) in the MPC group and 47.6 mL/m^2^ (IQR, 37.7-52.4 mL/m^2^) in the control group. As demonstrated in the 3D echocardiography data, there was no significant difference between the groups in changes in LV mass (*P* = .71) or LV ejection fraction (*P* > .99). These imaging data are presented in Table 3 and Figure 5. The 2D echocardiography data showed similar trends as the 3D measurements (Table E1).

**Table 3:** LV size and function from baseline to 12 months by 3D echocardiography, cardiac MRI, and cardiac catheterization, by treatment assigned

Parameter	n	Total, median (IQR)	MPC group, median (IQR)	Control group, median (IQR)	*P* value
3D echocardiography					
LVEDV indexed to BSA, mL/m^2^					.020
Baseline	14, 8, 6	19.9 (15.1-28.3)	17.4 (14.1-21.9)	27.1 (16.4-33.8)	
3 wk	6, 4, 2	24.9 (20.5-29.6)	29.2 (24.7-35.5)	16.3 (11.7-20.9)	
3-6 mo	2, 1, 1	30.3, 37.2	37.2	30.3	
12 mo	9, 3, 6	56.7 (41.4-81.7)	81.7 (56.7-107.8)	41.8 (37.3-60.8)	
LVESV indexed to BSA, mL/m^2^					.009
Baseline	14, 8, 6	7.8 (7.0-12.4)	7.6 (6.5-8.5)	14.0 (8.0-17.1)	
3 wk	6, 4, 2	8.2 (7.0-11.1)	9.8 (8.2-13.9)	5.5 (4.0-7.0)	
3-6 mo	2, 1, 1	10.6-13.8	13.8	10.6	
12 mo	9, 3, 6	20.8 (12.3-28.1)	28.1 (20.8-40.8)	12.9 (11.3-23.9)	
LV mass indexed to BSA, g/m^2^					.85
Baseline	14, 8, 6	19.1 (17.2-25.9)	18.5 (16.5-24.0)	20.6 (18.2-29.0)	
3 wk	6, 4, 2	21.0 (16.4-27.5)	25.5 (21.0-32.1)	13.7-16.4	
3-6 mo	2, 1, 1	19.5-35.2	35.2	19.5	
12 mo	8, 2, 6	32.4 (26.4-49.1)	28.3-64.4	32.4 (24.6-43.5)	
LV ejection fraction, %					.34
Baseline	14, 8, 6	56.0 (50.8-61.4)	58.7 (49.3-64.6)	52.6 (50.8-58.0)	
3 wk	7, 5, 2	66.3 (61.8-67.7)	63.0 (61.8-67.7)	66.3-67.0	
3-6 mo	2, 1, 1	62.8-65.0	62.8	65.0	
12 mo	9, 3, 6	65.6 (62.2-67.8)	63.0 (62.2-65.6)	67.4 (60.7-69.7)	
Cardiac MRI					
LVEDV indexed to BSA, mL/m^2^					.87
Baseline	19, 9, 10	17.7 (9.3-46.0)	15.7 (13.1-22.3)	17.8 (16.7-38.1)	
12 mo	16, 8, 8	47.3 (32.6-58.1)	40.4 (29.2-73.9)	47.6 (37.7-52.4)	
Difference	16, 8, 8	26.2 (16.0-35.3)	25.6 (16.5-49.6)	26.5 (16.0-31.1)	
LVESV indexed to BSA, mL/m^2^					.79
Baseline	19, 9, 10	8.0 (5.5-13.1)	6.3 (5.5-13.1)	8.9 (6.9-12.4)	
12 mo	16, 8, 8	13.6 (10.2-26.4)	14.3 (8.1-32.4)	13.6 (12.0-19.2)	
Difference	16, 8, 8	5.6 (4.4-14.6)	8.3 (3.3-18.1)	5.6 (5.4-10.0)	
LV mass indexed to BSA, g/m^2^					.71
Baseline	19, 9, 10	27.8 (18.2-31.0)	21.9 (19.2-30.7)	27.9 (17.6-31.0)	
12 mo	16, 8, 8	36.1 (28.9-47.5)	37.6 (31.3-47.5)	35.6 (27.4-44.7)	
Difference	16, 8, 8	9.9 (−1.0 to 19.6)	12.7 (−1.0 to 20.5)	8.9 (1.5-16.7)	
LV ejection fraction (%)					1.0
Baseline	19, 9, 10	56.3 (47.2-61.7)	57.2 (54.3-61.7)	54.5 (47.2-57.9)	
12 mo	16, 8, 8	65.8 (60.9-72.5)	64.5 (61.0-74.0)	65.8 (59.8-72.5)	
Difference	16, 8, 8	7.0 (−1.0 to 16.6)	7.0 (−0.6 to 12.9)	6.3 (−4.1 to 21.8)	
RV ejection fraction, %					.71
Baseline	19, 9, 10	58.0 (50.7-67.8)	58.0 (46.5-66.1)	57.8 (54.4-67.8)	
12 mo	16, 8, 8	53.5 (48.5-55.4)	54.1 (46.9-56.5)	51.7 (48.5-53.8)	
Difference	16, 8, 8	−4.8 (−14.4 to −2.9)	−4.6 (−14.0 to −1.7)	−5.5 (−16.0 to −2.9)	
Cardiac catheterization					
LVEDP, mmHg					.006
Baseline	17, 8, 9	12 (9-13)	9 (6.5-13.5)	12 (11-13)	
12 mo	15, 7, 8	13 (10-16)	16 (10-18)	12 (8.5-15.5)	
Difference	14, 7, 7	3 (0-5)	5 (4-8)	0 (−3 to 3)	
Mean LA pressure, mmHg					.68
Baseline	18, 9, 9	8.5 (8-9)	8 (8-9)	9 (8-9)	
12 mo	16, 8, 8	14 (12.5-15)	14 (12-15)	13.5 (12.5-14.5)	
Difference	15, 8, 7	5 (4-7)	5.5 (3.5-7.5)	5 (4-6)	

*IQR*, Interquartile range; *MPC*, mesenchymal precursor cell; *LVEDV*, left ventricular end-diastolic volume; *BSA*, body surface area; *LVESV*, left ventricular end-systolic volume; *LV*, left ventricular; *MRI*, magnetic resonance imaging; *RV*, right ventricular; *LA*, left atrial; *3D*, 3-dimensional.

**Figure E2:**
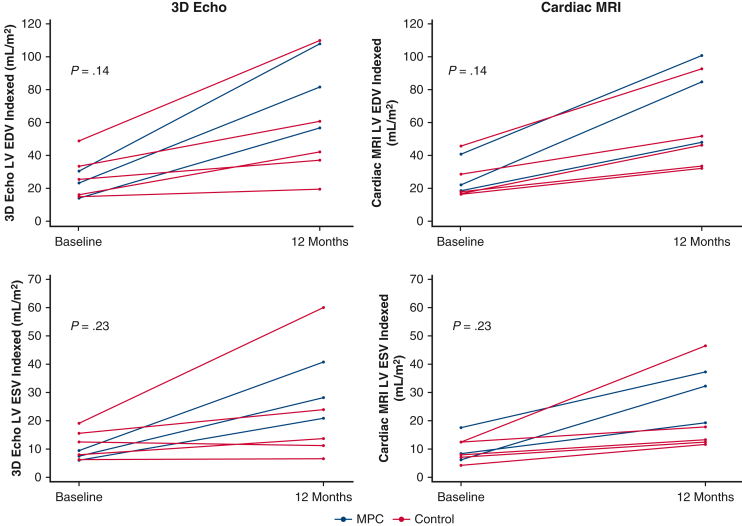
In the post hoc restricted analysis of 8 of 19 patients who had both 3-dimensional (*3D*) echocardiography and cardiac magnetic resonance imaging (*MRI*) at baseline and 12 months, the change in left ventricular end-diastolic volume (*LVEDV*) (*top*) and left ventricular end-systolic volume (*LVESV*) (*bottom*) from baseline to primary efficacy analysis at 1 year, by treatment assigned: 3D echocardiography (*left*) and cardiac MRI (*right*). *MPC*, Mesenchymal precursor cell.

However, when analyses were restricted only to patients who had both 3D echocardiography and cardiac MRI at baseline and 12 months, the median LVEDV at 12 months was nearly identical between the imaging modalities and was nominally larger in the MPC group (3D echocardiography: 81.7 mL/m^2^; cardiac MRI: 84.6 mL/m^2^) compared to the control group (3D echocardiography: 42.2 mL/m^2^; cardiac MRI: 46.4 mL/m^2^). Similar trends were observed for median LVESV (Table 4, Figure E2).

**Table 4:** LV size and function from baseline to 12 months by 3D echocardiography, cardiac MRI, and cardiac catheterization, by treatment assigned: restricted to patients who have both 3D echocardiography and cardiac MRI at baseline and 12 months

Parameter	n	Total, median (IQR)	MPC group, median (IQR)	Control group, median (IQR)	*P* value
3D echocardiography					
LVEDV indexed to BSA, mL/m^2^					.14
Baseline	8, 3, 5	24.6 (15.7-32.2)	23.4 (14.2-30.6)	25.9 (16.4-33.8)	
12 mo	8, 3, 5	58.7 (39.8-94.8)	81.7 (56.7-107.8)	42.2 (37.3-60.8)	
Difference	8, 3 ,5	34.8 (18.7-59.4)	58.2 (42.6-77.3)	25.8 (11.5-27.0)	
LVESV indexed to BSA, mL/m^2^					.23
Baseline	8, 3, 5	8.7 (6.9-14.0)	7.6 (5.9-9.4)	12.4 (8.0-15.6)	
12 mo	8, 3, 5	22.3 (12.5-34.4)	28.1 (20.8-40.8)	13.6 (11.3-23.9)	
Difference	8, 3, 5	11.6 (2.9-25.9)	20.5 (14.9-31.4)	5.6 (0.3-8.3)	
Cardiac MRI					
LVEDV indexed to BSA, mL/m^2^					.14
Baseline	8, 3, 5	20.6 (17.3-34.8)	22.3 (18.8-40.9)	17.9 (16.7-28.7)	
12 mo	8, 3, 5	50.0 (40.1-88.6)	84.6 (48.1-100.7)	46.4 (33.8-51.9)	
Difference	8, 3, 5	29.5 (19.6-53.2)	59.8 (29.3-62.3)	23.2 (16.0-29.8)	
LVESV indexed to BSA, mL/m^2^					.23
Baseline	8, 3, 5	8.0 (6.6-12.3)	8.2 (6.3-17.6)	7.7 (6.9-12.3)	
12 mo	8, 3, 5	18.6 (12.8-34.7)	32.2 (19.3-37.2)	13.2 (12.4-17.8)	
Difference	8, 3, 5	9.2 (5.5-22.8)	19.6 (11.1-26.0)	5.5 (5.4-7.4)	
Cardiac catheterization					
LVEDP, mmHg					.10
Baseline	8, 3, 5	12.5 (8.5-14)	8 (4-14)	13 (12-14)	
12 mo	8, 3, 5	11 (10-15.5)	10 (10-18)	12 (10-15)	
Difference	8, 3, 5	2 (−1.5 to 3.5)	4 (2-6)	0 (−3 to 2)	
Mean LA pressure, mmHg					1.0
Baseline	7, 3, 4	9 (6-9)	8 (6-9)	9 (7-9.5)	
12 mo	7, 3, 4	14 (14-15)	14 (14-14)	14.5 (11-15)	
Difference	7, 3, 4	6 (5-8)	6 (5-8)	6 (2-8)	

*IQR*, Interquartile range; *MPC*, mesenchymal precursor cell; *3D*, 3-dimensional; *LVEDV*, left ventricular end-diastolic volume; *BSA*, body surface area; *LVESV*, left ventricular end-systolic volume; *MRI*, magnetic resonance imaging; *LA*, left atrial; *LV*, left ventricular.

**Figure E3:**
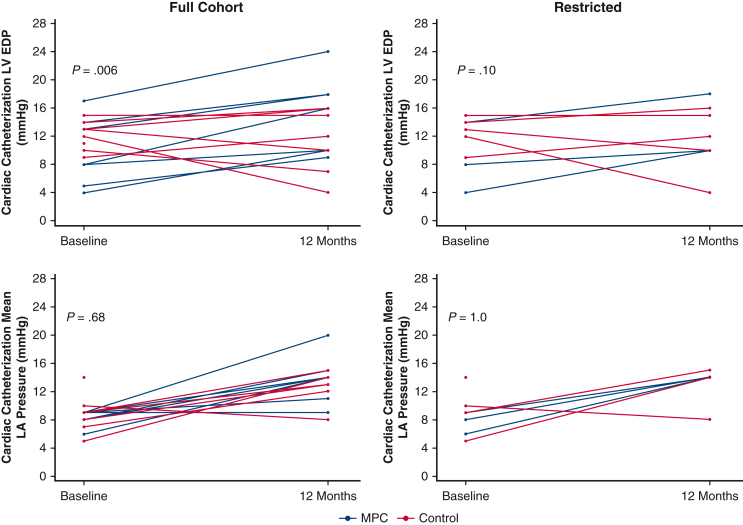
Cardiac catheterization data including left ventricular (*LV*) end-diastolic pressure (*top*) and mean left atrial (*LA*) pressure (*bottom*), by treatment assigned: full cohort analysis (*left*) and post hoc restricted analysis of 8 of 19 patients who had both 3-dimensional echocardiography and cardiac magnetic resonance imaging at baseline and 12 months (*right*). In the overall cohort, 15 of 19 patients had cardiac catheterization data. Missing data are not shown and did not contribute to median calculations and comparisons between groups. *MPC*, Mesenchymal precursor cell; *LV EDP*, left ventricular end diastolic pressure.

By cardiac catheterization, there was no difference between the groups in the change in mean left atrial (LA) pressures from baseline to 12 months in both the overall cohort and the restricted analysis (*P* = .68 and *P* > .99, respectively). The increases in LVEDP from baseline to 12 months were higher in the MPC group in the overall cohort (*P* = .006), but the difference was not significant in the restricted analysis (*P* = .10) (Tables 3 and 4, Figure E3).

## Discussion

Staged LV recruitment is feasible in patients with HLHS and borderline LV after single ventricle palliation, with >50% of patients in both the MPC and control groups undergoing BiV or 1.5V conversion within 2 years. This study used MPC injection in the hypoplastic LV as a safe and feasible adjunctive therapy to surgical LV recruitment. There were no SAEs deemed related to the trial and no differences in the frequency, timing, or severity of AEs between the groups. There was also minimal evidence of immunologic reaction to the MPC injections, including donor antigen sensitization, local tissue inflammation, arrhythmia, or tumor formation.

Preliminary analyses of LV size and function demonstrated a larger increase in LVEDV and LVESV in the MPC-treated group at the 1-year follow-up, suggesting potentially greater LV growth and remodeling in patients receiving MPCs. The fact that 100% of MPC-treated children compared with 57% of controls had large enough LVs to accommodate the full BiV conversion suggests that MPC treatment may help increase the ability to “better grow” the HLHS LV after LV recruitment surgery. However, this early-phase trial was designed to investigate safety and feasibility and was not powered to detect efficacy with an enrolled cohort of 19 patients.

Cardiac MRI and 3D echocardiography are preferred imaging modalities that have previously demonstrated comparability and accuracy in visualization of small and irregularly shaped LVs.^22^ In this trial, a majority of the patients (16 of 19) underwent cardiac MRI at both baseline and 12 months, whereas only roughly one-half of the patients (9 of 19) had 3D echocardiography with LV measurements at both time points. Thus, the differences between groups observed at 12 months by 3D echocardiography but not by cardiac MRI in the total cohort may represent differences in the subset of subjects who were able to obtain regular follow-up imaging. Indeed, in the post hoc analysis of only patients who underwent both imaging modalities at baseline and 12 months, there was a 2-fold larger increase in LVEDV and a 3-fold larger increase in LVESV in the MPC patients, which was consistent between imaging modalities. This subanalysis is limited by sample size but suggests that MPC-treated patients may have increased LV chamber remodeling at 12 months compared to controls, a finding that could translate into improved cardiac output and systemic perfusion in young children over time.

The inherent diversity of baseline anatomy and differences in LV recruitment maneuvers could introduce variability in blood flow to the hypoplastic LV, leading to differences in LV growth and likelihood of achieving 1.5V/BiV conversion but should balance out between the groups with randomization. In our trial, groups were balanced by chance with regard to LV recruitment maneuvers and HLHS versus unbalanced AV canal diagnosis; however, intergroup variability would be further reduced in larger randomized trials.

Our results are consistent with previous human and animal studies suggesting the feasibility of MLCs for cardiovascular disease in adult populations, including intracoronary administration following MI^8^, ^9^, ^10^, ^11^, ^12^ and transendocardial administration in patients with ischemic or nonischemic cardiomyopathy.^14^ Recently, there has been increasing consideration of the role of stem cells in CHD, including HLHS. One open-label trial of intracoronary stem cell injections resulted in improved ventricular function and volumes, reduced heart failure status, improved quality of life, and reduced cardiac fibrosis in HLHS patients undergoing stage 2 or stage 3 palliation surgery.^15^ Other studies of MLCs in HLHS patients with single ventricle physiology to preserve the function of the systemic ventricle are ongoing.^16^^,^^17^^,^^23^ Our pilot trial contributes to the growing body of knowledge supporting MLCs as a potential therapy to enhance beneficial LV remodeling in patients with HLHS and specifically focuses on a new and growing population of patients undergoing LV recruitment with the goal of BiV/1.5V conversion.

Future studies using animal and human models are needed to investigate the mechanism of action of MPCs in HLHS. In combination with LV recruitment surgeries, MLCs have been hypothesized to promote long-term myocardial elasticity, LV chamber growth, angiogenesis, and vitality. A major challenge to LV recruitment is the development of EFE, causing restrictive cardiomyopathy and preventing growth of the hypoplastic LV. Previous studies of MLCs in patients following MI have demonstrated that these cells inhibit cardiac fibrosis through secretion of antifibrotic hepatocyte growth factor, direct cellular interactions with cardiac myofibroblasts, and inhibition of profibrotic signaling.^24^^,^^25^ An LV biopsy protocol or novel noninvasive imaging technique could facilitate tracking of MPC location and durability following administration, contributing to an understanding of MPC mechanism. Our study used allogenic MPCs, which have been shown to be safe,^26^ have been trialed in pediatric populations with HLHS,^18^^,^^23^ and are more readily available. However, further investigations also could explore the use of other cell lineages, such as skeletal myocytes and cardiosphere-derived cells.

In the DREAM-HF trial of patients with heart failure with reduced ejection fraction, a single transendocardial administration of 150 × 10^6^ MPCs showed a significant reduction in the risk of MI or stroke in MPC-treated patients, with a stronger effect in patients with high baseline inflammation.^14^ Importantly, patients with HLHS have an inflammatory response following cardiopulmonary bypass and surgical interventions but also have high levels of preoperative inflammation.^27^ Additional research to understand the interaction between inflammation and MPC efficacy in HLHS is needed to inform which patients may benefit most from this therapy.

Allosensitization is a crucial issue in patients with HLHS, who may require cardiac transplantation later in life and often are frequently exposed to blood and other product administration in the setting of multiple cardiac surgeries. Thus, it is a priority to understand whether allogeneic MPCs could cause further sensitization in this vulnerable population. One trial of transendocardial injection of allogeneic versus autologous mesenchymal stem cells in 37 patients with nonischemic dilated cardiomyopathy found a significantly greater increase in PRA in patients who received allogeneic cells at 6 months, although notably, only 1 patient in the allogeneic group had antibodies against DSA.^28^ The population of patients in our study was highly sensitized, with a baseline PRA >90% in one-half of the patients. This might have hindered our ability to detect a further increase in PRA following MPC administration. Additionally, it is conceivable that high sensitization against DSA at baseline could lead to immune destruction of circulating MPCs, limiting therapeutic efficacy. However, the MPCs used in this study are hypoimmunogenic and should be well tolerated regardless of the degree of HLA-matching. There was minimal sensitization to donor antigens in our study, although our sample size was small. Moreover, there was no difference in BiV/1.5V conversion in patients with high versus low baseline HLA sensitization (data not shown). However, further studies could investigate the response to MLCs in populations with different levels of baseline sensitization. Specifically, future trials should include immunologic monitoring following MPC administration in a large cohort of unsensitized patients, better enabling detection of sensitization as a result of allogeneic MPC therapy.

The high rate of BiV/1.5V conversion in our cohort supports the potential of this staged BiV recruitment approach in select HLHS patients receiving care at well-equipped surgical centers. Some of the remaining patients with a single ventricle physiology were still under consideration for BiV conversion at final follow-up. As staged ventricular recruitment and BiV conversion becomes an increasingly viable strategy for select HLHS patients, MPC injections in hypoplastic LVs may hold increasing therapeutic potential. Having demonstrated safety and feasibility in this cohort, larger trials using dose-ranging designs powered to detect efficacy are crucial to further investigate the promising therapeutic potential of MPC injections in this complex patient population.

### Webcast

You can watch a Webcast of this AATS meeting presentation by going to: https://www.aats.org/resources/lb4-prospective-single-center-randomized-control-trial-to-evaluate-the-safety-and-feasibility-of-a-novel-allogenic-mesenchymal-precursor-cell-therapy-in-patients-with-hypoplastic-left-heart-syndrome-2.

**Figure:**
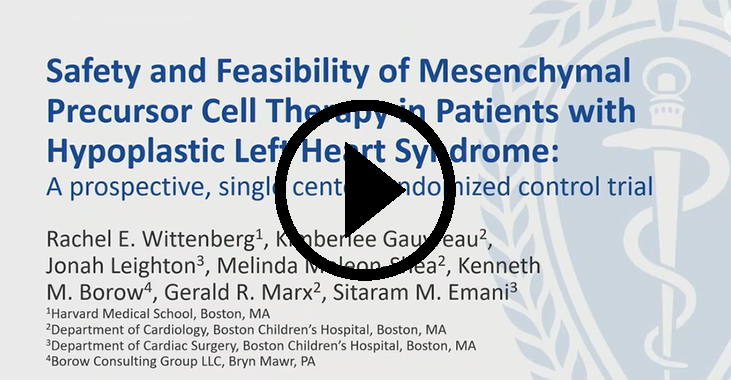

## Conflict of Interest Statement

K.M.B. is a consultant for Mesoblast. S.M.E. is a consultant for Chiesi Pharmaceuticals. All other authors reported no conflicts of interest.

The *Journal* policy requires editors and reviewers to disclose conflicts of interest and to decline handling or reviewing manuscripts for which they may have a conflict of interest. The editors and reviewers of this article have no conflicts of interest.

## Figures and Tables

**Table E1:** LV size and function from baseline to 24 months by 2D echocardiography, by treatment assigned

Parameter	N	Total (N = 19), median (IQR)	MPC (N = 9), median (IQR)	Control (N = 10), median (IQR)	*P* value
LVEDV indexed to BSA, mL/m^2^					.025
Baseline	14, 5, 9	21.8 (12.5-29.0)	13.5 (9.9-27.2)	26.3 (17.0-29.0)	
3 wk	9, 4, 5	29.8 (26.3-35.5)	27.0 (22.2-35.3)	30.8 (26.6-35.5)	
3-6 mo	6, 1, 5	38.7 (20.8-46.7)	45.9	31.6 (20.8-46.7)	
12 mo	15, 7, 8	43.8 (28.2-64.7)	49.2 (21.1-81.7)	40.7 (33.6-50.4)	
24 mo	6, 3, 3	46.4 (30.3-56.9)	56.9 (41.4-58.2)	30.3 (29.6-51.4)	
LVESV indexed to BSA, mL/m^2^					.044
Baseline	14, 5, 9	8.2 (5.2-15.8)	5.2 (4.8-8.7)	13.8 (7.7-16.1)	
3 wk	9, 4, 5	9.3 (7.3-16.4)	8.9 (6.7-13.4)	9.3 (7.9-23.9)	
3-6 mo	5, 1, 4	14.6 (6.1-18.8)	14.6	12.5 (4.8-22.8)	
12 mo	15, 7, 8	17.0 (11.3-26.1)	17.0 (8.3-28.1)	14.8 (11.9-21.2)	
24 mo	6, 3, 3	17.9 (13.5-21.6)	21.6 (14.2-23.3)	13.5 (7.6-21.5)	
LV mass indexed to BSA, g/m^2^					.44
Baseline	12, 3, 9	28.5 (20.0-36.6)	23.9 (14.8-27.6)	34.9 (20.4-36.7)	
3 wk	8, 4, 4	35.9 (31.9-51.3)	35.8 (27.6-42.4)	44.8 (32.1-56.4)	
3-6 mo	7, 2, 5	41.1 (37.4-64.7)	61.1-71.7	38.5 (37.4-41.1)	
12 mo	13, 6, 7	40.5 (32.8-50.9)	39.2 (23.8-59.7)	40.5 (38.9-50.9)	
24 mo	6, 3, 3	40.6 (32.4-46.4)	41.7 (28.0-46.4)	39.4 (32.4-54.3)	
LV ejection fraction, %					.99
Baseline	14, 5, 9	54.8 (44.6-60.1)	57.6 (54.0-64.0)	49.0 (44.6-55.9)	
3 wk	11, 5, 6	66.0 (56.8-74.2)	70.0 (60.1-71.0)	65.5 (55.7-74.2)	
3-6 mo	9, 3, 6	74.2 (68.3-80.0)	71.0 (68.3-80.0)	75.1 (59.6-81.0)	
12 mo	15, 7, 8	63.0 (54.2-68.0)	65.5 (55.7-65.6)	60.6 (53.7-70.8)	
24 mo	6, 3, 3	61.0 (58.1-65.8)	62.0 (60.0-65.8)	58.1 (55.6-74.7)	

*IQR*, Interquartile range; *MPC*, mesenchymal precursor cell; *LVEDV*, left ventricular end-diastolic volume; *BSA*, body surface area; *LVESV*, left ventricular end-systolic volume; *LV*, left ventricular; *2D*, 2-dimensional.

## References

[1] Martino D., Rizzardi C., Vigezzi S., Guariento C., Sturniolo G., Tesser F. (2022). Long-term management of Fontan patients: the importance of a multidisciplinary approach. Front Pediatr.

[2] Hickey E.J., Caldarone C.A., Blackstone E.H., Lofland G.K., Yeh T., Pizarro C. (2007). Critical left ventricular outflow tract obstruction: the disproportionate impact of biventricular repair in borderline cases. J Thorac Cardiovasc Surg.

[3] Chiu P., Emani S. (2021). Left ventricular recruitment in patients with hypoplastic left heart syndrome. Semin Thorac Cardiovasc Surg Pediatr Card Surg Annu.

[4] Prasanna A., Tan C.W., Anastasopulos A., Beroukhim R.S., Emani S.M. (2022). One and one-half ventricle repair: role for restricting antegrade pulmonary blood flow. Ann Thorac Surg.

[5] Emani S.M., McElhinney D.B., Tworetzky W., Myers P.O., Schroeder B., Zurakowski D. (2012). Staged left ventricular recruitment after single-ventricle palliation in patients with borderline left heart hypoplasia. J Am Coll Cardiol.

[6] Psaltis P.J., Paton S., See F., Arthur A., Martin S., Itescu S. (2010). Enrichment for STRO-1 expression enhances the cardiovascular paracrine activity of human bone marrow-derived mesenchymal cell populations. J Cell Physiol.

[7] See F., Seki T., Psaltis P.J., Sondermeijer H.P., Gronthos S., Zannettino A.C. (2011). Therapeutic effects of human STRO-3-selected mesenchymal precursor cells and their soluble factors in experimental myocardial ischemia. J Cell Mol Med.

[8] Mathur A., Arnold R., Assmus B., Bartunek J., Belmans A., Bönig H. (2017). The effect of intracoronary infusion of bone marrow-derived mononuclear cells on all-cause mortality in acute myocardial infarction: rationale and design of the BAMI trial. Eur J Heart Fail.

[9] Roncalli J., Mouquet F., Piot C., Trochu J.N., Le Corvoisier P., Neuder Y. (2011). Intracoronary autologous mononucleated bone marrow cell infusion for acute myocardial infarction: results of the randomized multicenter BONAMI trial. Eur Heart J.

[10] Choudry F., Hamshere S., Saunders N., Veerapen J., Bavnbek K., Knight C. (2016). A randomized double-blind control study of early intra-coronary autologous bone marrow cell infusion in acute myocardial infarction: the REGENERATE-AMI clinical trial†. Eur Heart J.

[11] Wöhrle J., von Scheidt F., Schauwecker P., Wiesneth M., Markovic S., Schrezenmeier H. (2013). Impact of cell number and microvascular obstruction in patients with bone-marrow derived cell therapy: final results from the randomized, double-blind, placebo controlled intracoronary Stem Cell therapy in patients with Acute Myocardial Infarction (SCAMI) trial. Clin Res Cardiol.

[12] Sürder D., Manka R., Moccetti T., Lo Cicero V., Emmert M.Y., Klersy C. (2016). Effect of bone marrow-derived mononuclear cell treatment, early or late after acute myocardial infarction: twelve months CMR and long-term clinical results. Circ Res.

[13] Tompkins B.A., Rieger A.C., Florea V., Banerjee M.N., Natsumeda M., Nigh E.D. (2018). Comparison of mesenchymal stem cell efficacy in ischemic versus nonischemic dilated cardiomyopathy. J Am Heart Assoc.

[14] Perin E.C., Borow K.M., Henry T.D., Mendelsohn F.O., Miller L.W., Swiggum E. (2023). Randomized trial of targeted transendocardial mesenchymal precursor cell therapy in patients with heart failure. J Am Coll Cardiol.

[15] Ishigami S., Ohtsuki S., Eitoku T., Ousaka D., Kondo M., Kurita Y. (2017). Intracoronary cardiac progenitor cells in single ventricle physiology: the PERSEUS (cardiac progenitor cell infusion to treat univentricular heart disease) randomized phase 2 trial. Circ Res.

[16] Efficacy and safety study of autologous cardiac stem cells (JRM-001) treated after reconstructive surgery in pediatric patients with congenital heart disease: a multicenter randomized single-blind parallel-group study. ClinicalTrials.gov identifier: NCT02781922. NCT02781922.

[17] Nelson T.J. Phase I safety and feasibility study of intracoronary delivery of autologous bone marrow derived mononuclear cells for systemic, single right ventricular failure due to congenital heart disease. ClinicalTrials.gov identifier: NCT02549625. NCT02549625.

[18] Kaushal S., Hare J.M., Hoffman J.R., Boyd R.M., Ramdas K.N., Pietris N. (2023). Intramyocardial cell-based therapy with Lomecel-B during bidirectional cavopulmonary anastomosis for hypoplastic left heart syndrome: the ELPIS phase I trial. Eur Heart J Open.

[19] Ascheim D.D., Gelijns A.C., Goldstein D., Moye L.A., Smedira N., Lee S. (2014). Mesenchymal precursor cells as adjunctive therapy in recipients of contemporary left ventricular assist devices. Circulation.

[20] Yau T.M., Pagani F.D., Mancini D.M., Chang H.L., Lala A., Woo Y.J. (2019). Intramyocardial injection of mesenchymal precursor cells and successful temporary weaning from left ventricular assist device support in patients with advanced heart failure: a randomized clinical trial. JAMA.

[21] Perin E.C., Borow K.M., Silva G.V., DeMaria A.N., Marroquin O.C., Huang P.P. (2015). A phase II dose-escalation study of allogeneic mesenchymal precursor cells in patients with ischemic or nonischemic heart failure. Circ Res.

[22] Friedberg M.K., Su X., Tworetzky W., Soriano B.D., Powell A.J., Marx G.R. (2010). Validation of 3D echocardiographic assessment of left ventricular volumes, mass, and ejection fraction in neonates and infants with congenital heart disease: a comparison study with cardiac MRI. Circ Cardiovasc Imaging.

[23] Kaushal S., Wehman B., Pietris N., Naughton C., Bentzen S.M., Bigham G. (2017). Study design and rationale for ELPIS: a phase I/IIb randomized pilot study of allogeneic human mesenchymal stem cell injection in patients with hypoplastic left heart syndrome. Am Heart J.

[24] Li X., Zhao H., Qi C., Zeng Y., Xu F., Du Y. (2015). Direct intercellular communications dominate the interaction between adipose-derived MSCs and myofibroblasts against cardiac fibrosis. Protein Cell.

[25] Kishore R., Verma S.K., Mackie A.R., Vaughan E.E., Abramova T.V., Aiko I. (2013). Bone marrow progenitor cell therapy-mediated paracrine regulation of cardiac miRNA-155 modulates fibrotic response in diabetic hearts. PLoS One.

[26] Hare J.M., Fishman J.E., Gerstenblith G., DiFede Velazquez D.L., Zambrano J.P. (2012). Comparison of allogeneic vs autologous bone marrow–derived mesenchymal stem cells delivered by transendocardial injection in patients with ischemic cardiomyopathy: the POSEIDON randomized trial. JAMA.

[27] Appachi E., Mossad E., Mee R.B.B., Bokesch P. (2007). Perioperative serum interleukins in neonates with hypoplastic left-heart syndrome and transposition of the great arteries. J Cardiothorac Vasc Anesth.

[28] Hare J.M., DiFede D.L., Rieger A.C., Florea V., Landin A.M., El-Khorazaty J. (2017). Randomized comparison of allogeneic versus autologous mesenchymal stem cells for nonischemic dilated cardiomyopathy. J Am Coll Cardiol.

